# A methodology for on‐board CBCT imaging dose using optically stimulated luminescence detectors

**DOI:** 10.1120/jacmp.v17i5.6378

**Published:** 2016-09-08

**Authors:** Noor Mail, Muhammad Yusuf, Nazeeh Alothmany, A. Abdulrahman Kinsara, Fahad Abdulkhaliq, Suliman M. Ghamdi, Abdelhamid Saoudi

**Affiliations:** ^1^ King Abdullah International Medical Research Center (KAIMRC) Jeddah Saudi Arabia; ^2^ Princess Norah Oncology Center, National Guard Health Affairs Jeddah Saudi Arabia; ^3^ Electrical and Computer and Engineering Department King Abdulaziz University Jeddah Saudi Arabia; ^4^ Department of Nuclear Engineering Center of Training and Radiation Protection, King Abdulaziz University Jeddah Saudi Arabia

**Keywords:** OSLD calibration, body phantom, CBDI, point‐dose profiles, skin dose

## Abstract

Cone‐beam computed tomography CBCT systems are used in radiation therapy for patient alignment and positioning. The CBCT imaging procedure for patient setup adds substantial radiation dose to patient's normal tissue. This study presents a complete procedure for the CBCT dosimetry using the InLight optically‐stimulated‐luminescence (OSL) nanoDots. We report five dose parameters: the mean slice dose ($D_{\text{MSD}}$); the cone beam dose index (${\text{CBDI}}_{W}$); the mean volume dose ($D_{\text{MVD}}$); point‐dose profile, D(FOV); and the off‐field Dose. In addition, CBCT skin doses for seven pelvic tumor patients are reported. CBCT‐dose measurement was performed on a custom‐made cylindrical acrylic body phantom (50 cm length, 32 cm diameter). We machined 25 circular disks (2 cm thick) with grooves and holes to hold OSL‐nanoDots. OSLs that showed similar sensitivities were selected and calibrated against a Farmer‐type ionization‐chamber (0.6 CT) before being inserted into the grooves and holes. For the phantom scan, a standard CBCT‐imaging protocol (pelvic sites: 125 kVp, 80 mA and 25 ms) was used. Five dose parameters were quantified: $D_{\text{MSD}}$, ${\text{CBDI}}_{W}$, $D_{\text{MVD}}$, D(FOV), and the off‐field dose. The $D_{\text{MSD}}$ for the central slice was 31.1±0.85 mGy, and ${\text{CBDI}}_{W}$ was 34.5±0.6 mGy at 16 cm FOV. The $D_{\text{MVD}}$ was 25.6±1.1 mGy. The off‐field dose was 10.5 mGy. For patients, the anterior and lateral skin doses attributable to CBCT imaging were 39.04±4.4 and 27.1±1.3 mGy, respectively.

OSL nanoDots were convenient to use in measuring CBCT dose. The method of selecting the nanoDots greatly reduced uncertainty in the OSL measurements. Our detailed calibration procedure and CBCT dose measurements and calculations could prove useful in developing OSL routines for CBCT quality assessment, which in turn gives them the property of high spatial resolution, meaning that they have the potential for measurement of dose in regions of severe dose‐gradients.

PACS number(s): 87.57.‐s, 87.57.Q, 87.57.uq

## I. INTRODUCTION

One of the main objectives in radiation therapy is to deliver a uniform dose to the tumor whilst avoiding the organs at risk. Cone‐beam computed tomography (CBCT), provides a three‐dimensional image of the tumor, allowing the position of the patient to be adjusted prior to the start of the patient's treatment.

However, CBCT also delivers a small dose to healthy tissue around the target area, and as high CBCT doses have been reported in the literature,^1^, ^2^, ^3^, ^4^, ^5^, ^6^ each dose should be optimized and monitored for conformance with the ALARA principle. Palm et al.^(1)^ reported that doses from the OBI (v1.3) Varian system (Varian Medical Systems, Palo Alto, CA) can vary between 64 and 144 mGy and the dose from the v1.4 system between 1 and 51 mGy. The authors measured the dose with a thermoluminescent dosimeter (TLD) and a computed tomography (CT) dose profiler. Another study^(2)^ used an Elekta X‐ray volume imaging (XVI) system_(Elekta AB, Stockholm, Sweden) to calculate the CT dose index (${\text{CTDI}}_{W}$, a standardized measure of the radiation dose output of a CT scanner), and showed that the CBCT dose varied from 1.8 to 3.5 cGy. A TLD study by Wen et al.^(3)^ revealed a relationship between patient size and anterior–posterior skin dose and between dose and location on the body. The skin dose readings varied from 2.6 to 11 cGy. Song et al.^(5)^ evaluated and compared CBCT doses for XVI and OBI CBCT systems using a Farmer‐type ion chamber following the point‐dose measurements. Furthermore, they derived and reported linear relationships between the central axis dose and the total mAs used for each protocol setting. Nakonechny et al.^(7)^ measured the longitudinal single scan dose profile (SSDP) for several slice widths using a PTW diamond detector (PTW‐Freiburg GmbH, Freiburg, Germany) placed in a water‐equivalent plastic phantom. They qualitatively studied the effects of phantom shape, length, and composition on the measured SSDP.

The conventional method of measuring ${\text{CTDI}}_{W}$ relies on a single‐slice CT scanner with a pencil chamber of 100 mm length. The measured dose is referred to as ${\text{CTDI}}_{100}$ and represents the integral dose profile for a single slice. These types of CT chambers are widely used in clinics. However, in modern multislice CT scanners, the pencil chamber can underestimate the equilibrium dose and the dose line integral by 20%, as demonstrated when tested on body phantoms.^(8)^ The pencil chamber method of determining ${\text{CTDI}}_{100}$ is therefore not adequate for multislice scanners^(9)^ such as CBCT.

Accurately determining CBCT dose is therefore a research priority. One proposed solution was to make the ionization chamber longer than 100 mm in order to collect a wider tail of scattered radiation. CBCT scan uses large field size where the scattered beam is higher at the center slice location (mid field). The 100 mm chamber (or longer) may not be able to measure the exact dose at the central slice but can give only the mean dose from 10 cm length. It is convenient to use the small chamber (in length ∼2 cm) for the point‐dose measurement or the ${\text{CBDI}}_{W}$ for the central slice in a body phantom,^(8)^ but difficult to measure the off‐center doses or mean volume dose ($D_{\text{MVD}}$) with the Farmer ion chamber. Researchers have employed a variety of point‐dose detectors to measure ${\text{CTDI}}_{W}$, including small ion chambers,^(5)^ TLD,^(10)^ and the newer technique of optically stimulated luminescence (OSL).^11^, ^12^ OSL has been widely used in radiation protection and is being developed for dosimetry purposes in radiation therapy and diagnostic imaging.^13^, ^14^, ^15^, ^16^ To date, OSL has been proven to have a reliable clinical performance in both radiation therapy^(17)^ and diagnostic imaging.^11^, ^12^, ^18^, ^19^, ^20^, ^21^ OSL response is very sensitive in the diagnostic low energy range (15–150 kVp). Hypothetically, the average energy of the X‐ray spectrum is expected to increase as a function of depth due to the absorption of the low‐energy primary beam, but it can be compensated by the increase in Compton scatter (which has lower energy than the primary). There is a need for investigation of this energy sensitivity issue in CBCT, which guides us towards a consistent practical procedure for the calibration of OSL detectors in CBCT dosimetry.

Here, we report a comprehensive procedure for OSL nanoDots calibration and explain OSL dependence with depth. In addition, we investigate whether calibration at a single depth is sufficient for CBCT dosimetry. We provide, for the first time, a detailed evaluation of CBCT doses with respect to five parameters and their details, and how scatter affects the results. The parameters reported are 1) the mean slice dose, $D_{\text{MSD}}$, 2) the cone beam dose index, ${\text{CBDI}}_{W}$, 3) the mean volume dose, $D_{\text{MVD}}$, 4) the point dose profile, D(FOV), and 5) the off‐field dose. The point‐dose profiles of the body phantoms are presented along the superior‐inferior direction on the surface, at the center, and at several depths, within and outside the field of view (FOV). Additionally, CBCT skin doses at anterior and lateral locations for seven pelvic tumor patients are presented. It is worth mentioning that this study does not recommend the use of $D_{\text{MSD}}$ for periodic QA purposes, as $D_{\text{MSD}}$ calculation procedure is more time‐consuming, but it definitely does give us a fundamental knowledge and understanding regarding the level of approximation and accuracy in ${\text{CBDI}}_{W}$.

## II. MATERIALS AND METHODS

### A. CBCT Varian OBI system

We measured the kV CBCT dose for the Varian OBI system (version 1.4) on a Trilogy linear accelerator (Varian) using OSL nanoDot detectors. The details of the OBI system have been described extensively elsewhere.^(5)^ In brief, the system consists of an X‐ray tube capable of producing X‐ray beams with a peak energy range of 40–125 kV, and an image receptor at 140–170 cm from the source with a 100‐cm isocenter. CBCT images can be acquired in the full‐fan or half‐fan mode. In half‐fan mode, a half‐bowtie filter was used. This study used the standard CBCT imaging protocol for the pelvic region (125 kVp, 80 mA, and 25 ms) and a half‐fan beam acquisition with a half‐bowtie filter.

### B. Optically stimulated luminescent nanoDots

We used a commercially available OSL dosimetry system (Landauer Inc., Stillwater, OK) consisting of a small reader (microStar) and a detector (InLight nanoDot). NanoDots are comprised of a radiation‐sensitive material (4 mm diameter, 0.2 mm thick) enclosed in a light‐tight envelope with dimensions $10\times 10\times 2\;{\text{mm}}^{3}$. Carbon‐doped aluminum oxide (${\text{Al}}_{2}O_{3}:C$) is used, primarily because of its high sensitivity to radiation (40–60 times that of LiF (TLD‐100)). It has a principal emission peak between 410 and 420 nm (blue). It operates over a wide energy range, from 5 keV to 20 MeV, with energy dependence within ±10% for a diagnostic range of 70‐140 keV and within ±5% for photons and electrons of 5–20 MeV.^(15)^ In the case of CBCT at 125 kV (HVL of 6 mm Al), its effective energy would be somewhere between 45 and 48 keV. The system has a useful dose ranging from 10 μGy to >100 Gy, with a linear response up to 3 Gy. The OSLDs used in this study had no angular dependence in the range from 80 to 125 kV. For more details see Yusuf et al.^(22)^

### C. Acrylic phantoms for dose measurements

**Figure 1:**
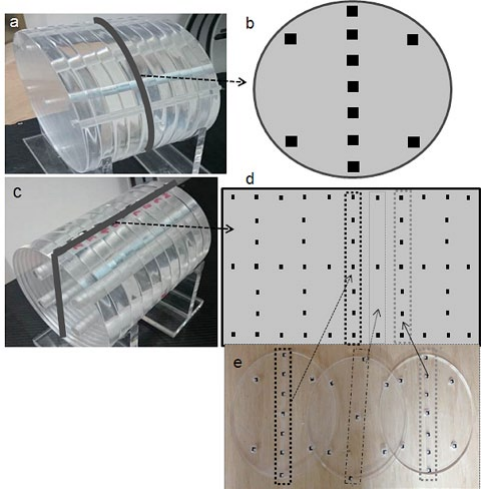
The custom‐made Lucite body phantom: (a) an overall view; (b) schematic of the axial view of a body phantom disk, the arrow indicating its position in the phantom; (c) phantom with OSL nanoDots placed in the indicated plane; (d) schematic of the nanoDots placed in the phantom, the arrow indicating the plane location in the phantom; (e) the three slabs with OSLDs, the arrows indicating the locations of the slabs and OSLDs in the schematic (d).

We machined 2‐cm‐thick acrylic into circular disks of 32 cm diameter, as shown in Fig. 1(a). These disks were stacked to make phantoms of varying length. Each disk had four peripheral holes, and one central hole into which the nanoDots were placed. Any holes that were not filled by nanoDots were filled by acrylic rods. Some disks were grooved along their diameter and a line of nanoDots assembled in a line along each grove, to allow us to insert nanoDots within a phantom at specific distances from the radiation source. The spaces between the nanoDots were approximately 2–3 cm and filled by acrylic cylinders along the superior–inferior direction and rectangular slabs ($0.2\times 1\times 2\;{\text{cm}}^{3}$) along the diameter of the body phantom. Some of the placement positions of the OSL nanoDots are presented in Fig. 1, which shows 20 disks (40 cm). This was sufficient to collect the scattered dose outside the radiation field, because our maximum scanning length (i.e., our FOV) was 16 cm. The off‐field (outside the FOV) measurements were recorded at several distances from the field edges.

### D. Determination of OSL calibration factor

We irradiated 500 nanoDots from a single batch in 10 separate exposures at 125 kVp. In each exposure, 50 NanoDots were placed on the Lucite slab in a rectangular area of $5\times 10\;{\text{cm}}^{2}$, where 5 cm was along the lateral and 10 cm was along the superior–inferior direction to minimize the heel effect. Approximately 100 nanoDots with a 1%–2% variation were selected. A 0.6 CT Farmer‐type ionization chamber (Radcal, Monrovia, CA) was used with the OSLD. The ion chamber was calibrated in air by the Radcal‐accredited laboratory (directly traceable to NIST) at beam quality M150 (150 kVp, HVL 10.2 mm Al) listed in Task group (TG) 61.^(23)^ The in‐phantom calibration procedure described in TG 61 was followed, as

(1) $$D_{w,z=2cm}=MN_{k}P_{Q,\text{cham}}P_{\text{sheath}}{\left[{\left({\bar{\mu_{en}}}_{/\rho }\right)}_{\text{air}}^{w}\right]}_{\text{water}}$$

Equation (1) was slightly modified for the Lucite phantom, as

(2) $$D_{w,z=2cm}=MN_{k}P_{Q}P_{\text{cham}}{\left[{\left({\bar{\mu_{en}}}_{/\rho }\right)}_{\text{air}}^{w}\right]}_{\text{med}}$$

where *M* is the chamber reading (corrected for temperature, pressure, recombination, and polarity effect), with the center placed exactly at 2 cm depth in the phantom, $N_{k}$ the air‐kerma calibration factor for the given beam quality (M150). The correction factor for the waterproofing sleeve, $P_{\text{sheath}}=1$ in the solid phantom. The factor $P_{Q,\text{cham}}(=P_{Q}\times P_{\text{cham}})$ was split into beam quality factor $P_{Q}$ and the $P_{\text{cham}}$, responsible for the angular distribution of the photon beam in the phantom compared to that used for the calibration in air. $P_{Q}(=1.007)$ was calculated for the user beam (125 kVp, HVL 6 mm Al) from the ion chamber response table provided by the Radcal at various beam qualities. The value of $P_{\text{cham}}(=1.008)$ was calculated from TG‐61 with the assumption that the difference of $P_{Q,\text{cham}}$ between Lucite and water is neglig ${\left({\bar{\mu }}_{en{/}_{\rho }}\right)}_{\text{air}}^{m}$ is the ratio for the Lucite‐to‐air of the mean mass energy‐absorption coefficients averaged over the photon spectrum at the calibration depth. Three nanoDots were calibrated against an ionization chamber at 125 kVp that had a reproducible output with ±1% variation. The nanoDots were used to perform CBCT dose measurements. The calibration factors for the nanoDots were obtained at different depths, from the surface to 16 cm deep, as explained below.

**Figure 2:**
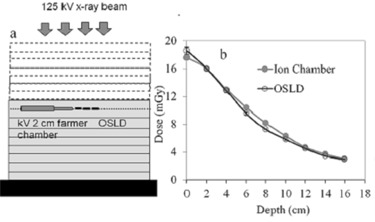
Schematic (a) of the OSLD calibration against kV ionization chamber (0.6 CT, Radcal). Dose (b) as a function of depth in the Lucite phantom at 125 kVp measured with OSLD and ion chamber. The OSLD calibration at a depth of 2 cm against the ionization chamber is compared with the OSLD calibration against the ion chamber at all depths. The variation between the OSL nanoDots and ion chamber is less than 8.5%.

The calibration setup for the OSLD against the ionization chamber is shown in Fig. 2(a). The ionization chamber and the nanoDots were placed on the same plane, 2 cm apart. Two Lucite slabs ($30\times 30\times 2\;{\text{cm}}^{3}$) were machined to make trenches of size $140\times 10\times 1\;{\text{mm}}^{3}$. The machined Lucite slabs were glued with each other in such a way that the trench of one slab was facing the trench of the other. A hole (150 mm long and 12 mm in diameter) was drilled for the Farmer ionization chamber (with a Lucite buildup cap diameter of 12 mm) as shown in Fig. 2(a). Later on, the slabs were separated for convenience in placing the OSLDs in the trench. Empty spaces of the remaining trench were filled with Lucite strips. Three OSLDs were placed in line without any air gaps. The ion chamber and the OSLDs were placed along superior–inferior direction to avoid the heel effect. 16‐cm‐thick Lucite slabs were placed underneath the ion chamber and the OSLD to provide a full scatter condition. Source‐to‐ion chamber/OSLD distance was kept to 100 cm throughout the calibration procedure. Dose readings were taken from 0 to 16 cm deeper in the phantom by adding Lucite slabs with equivalent thickness of 2 cm on the top after each reading. Exposure in the air at 100 cm was kept the same. All exposure measurements were normalized by using a second identical chamber (0.6 CT, Radcal) in air to avoid the fluence variation from the X‐ray source. In this study, the dose measured from the nanoDot dosimeters after kV‐CBCT irradiation ($D_{\text{OSLD}}$) can be expressed as:

(3) $$D_{\text{OSLD}}^{m}=M_{\text{OSLD}}^{m}(d)\times F(d)\times \frac{R_{\text{Ioncham}}^{\text{AIR}}(R1)}{R_{\text{Ioncham}}^{\text{AIR}}(Rn)}$$

where $R_{\text{Ioncham}}^{\text{AIR}}(Rl)$ and $R_{\text{Ioncham}}^{\text{AIR}}(Rn)$ are the first and n‐th reading of the ion chamber in air, *F*(*d*) is the depth correction factor (for the variation in energy response), and $M_{\text{OSLD}}(d)$ is the reading with OSL dosimeter at depth d in the Lucite phantom for any exposure. The index m refers to Lucite material. The ratio R1/Rn in Eq. (3) was used to correct for the source or exposure variation. F(d) is the ratio of the dose measured with the ionization chamber ($D_{\text{ionchamber}}$) to that of the OSLD reading ($M_{\text{Cal OSL}}(d)$) recorded during calibration as a function of depth in the phantom,

(4) $$F(d)=\frac{D_{\text{Ioncham}}^{m}(d)}{M_{\text{CalOSL}}^{m}(d)}$$

Because calibration against the ionization chamber involved more work, a single‐point calibration at a 2 cm depth against the ionization chamber was also tested. For this single‐point calibration, Eq. (3) was modified as follows:

(5) $$D_{\text{OSLD}}^{m}=M_{\text{OSLD}}^{m}(d)\times \frac{D_{\text{Ioncham}}^{m}(2\;cm)}{M_{\text{CalOSL}}^{m}(2\;cm)}\times \frac{R_{\text{Ioncham}}^{\text{AIR}}(R1)}{R_{\text{Ioncham}}^{\text{AIR}}(Rn)}$$

where $D_{\text{ionchamber}}(2cm)$ and $M_{\text{OSLD}}(2cm)$ are the doses measured at the 2‐cm depth in the Lucite phantoms by the ionization chamber and OSL dosimeter, respectively. The calibration factor was determined by placing nanoDot dosimeters at the location where the dose was measured by the calibrated ionization chamber. The CBCT scan required projections to be collected across 360° of rotation using the cylindrical Lucite phantom. The calibration factor defined in Eq. (5) was tested at different depths for the CBCT scans against the ionization chamber.

### E. CBCT scans

In the first configuration, 11 nanoDots were placed on the phantom surface, equally spaced 2 cm apart; Fig. 1(c)). In a second configuration, 11 nanoDots were placed at peripheral positions, in the grooves along the superior–inferior direction. In a third, 11 nanoDots were placed along the central axis of the phantom. The gaps were filled by acrylic rods, as shown in Fig. 1(d). Seven nanoDots were placed along the rims of the phantoms on the central acrylic slab, which had been grooved to hold the nanoDots in place, as shown in Fig. 1(b).

With the nanoDots in place, the phantoms were scanned on the Varian OBI CBCT system, using a half‐bowtie filter at 125 kVp, a current of 80 mA, and an exposure time of 25 ms per projection. The number of projections was 650 per half‐fan CBCT scan. The phantoms were scanned at 5, 8, 12, and 16 cm FOV. There were 11 measurement points (nanoDots) on the surface and central axis, covering 20 cm of the 40 cm total length of the phantom. At least two nanoDots were placed outside the FOV to measure the scattered dose outside the scanning length. All nanoDots were collected and read by the reader for each FOV (or scanning length).

The calibration factor was applied to the data. The error bars were obtained from the standard deviation of the OSLD reading after the application of the depth correction. The measured point doses at the phantom's center, periphery, and surface were plotted as a function of FOV. An exponential mathematical equation was used to fit the point dose data,

(6) $$D=A\times \left[1-\text{EXP}\left(-\frac{x}{B}\right)\right]$$

where *x* represents the scanning length, and *A* and *B* are the fitting curve constants.

### F. CBDI with OSLD

CBCT uses a wider beam, and a larger area can be imaged with a single scan, in a shorter duration. The dose in the CBCT projection is expected to be higher at the center of the FOV, or central slice, because the phantom's scatter is higher at the center. The OSLDs were placed at the center and four peripheral locations in the custom‐made body phantom shown in Fig. 1(b). Measurements at the center and periphery were recorded as the ${\text{CBDI}}_{\text{center}}$ and ${\text{CBDI}}_{\text{periphery}}$, respectively. The ${\text{CBDI}}_{W}$ for the central slice in the mid‐field was calculated as

(7) $${\text{CBDI}}_{w}=\frac{1}{3}{\text{CBDI}}_{\text{center}}+\frac{2}{3}{\text{CBDI}}_{\text{periphery}}$$

### G. Mean slice dose and mean volume dose

The ${\text{CBDI}}_{W}$ calculation in Eq. (7) is an approximation of the average dose for a single slice taken at two points (periphery and center). The average (mean) slice dose, $D_{\text{MSD}}$, can be more accurately calculated using OSL nanoDot data via the following equation:

(8) $$D_{\text{MSD}}=\frac{\int_{l}dz\int_{-R}^{R}2\pi r(Ar^{2}+Br+C)dr}{\pi R^{2}l}$$

where *l* is the slice thickness and *D*(*r*) is the fitting equation of the measured dose profile along the diameter of the slice. As the nanoDots record the point dose within the slice thickness (with almost no variation expected along the z‐axis within a slice thickness of 2.5 mm) Eq. (8) can be further simplified as:

(9) $$D_{\text{MSD}}=\frac{\int_{-R}^{R}2\pi rD(r)dr}{\pi R^{2}}=\frac{2\pi \int_{-R}^{R}r(Ar^{2}+Br+C)dr}{\pi R^{2}}$$

where *A, B*, and *C* are the fitting constants. $D_{\text{MSD}}$ was calculated for four different FOVs: 5, 8, 12, and 16 cm. $D_{\text{MSD}}(z)$ (mean slice dose as a function of slice or z) was calculated for 7 slices within the FOV or scanning length shown in Fig. 1(d), which is more illustrated in Fig. 1(e).

The $D_{\text{MSD}}$ for the rest of the slices were obtained from the fitting equation, called $D_{\text{MSD}}(z)$. In order to calculate the mean dose of the irradiated body volume, we needed to integrate $D_{\text{MSD}}(z)$ as a function of z (superior–inferior direction):

(10) $$D_{\text{MVD}}=\frac{\int_{0}^{V}D(r)dV}{\int_{0}^{V}dV}=\frac{\int_{-Z}^{Z}D_{\text{MSD}}(z)dz}{\int_{-Z}^{Z}dz}=\frac{\int_{-Z}^{Z}(C_{1}z^{2}+C_{2}z+C_{0})dz}{2Z}$$

where *D*(*r*) is the point dose at position *r* in an irradiated volume, *V*, and $D_{\text{MSD}}(z)$ is the slice dose (from the data fitting curve) as a function of *z* (superior–inferior direction) and *2Z* is the scanning length. [Note: It is important to mention here that the mean volume dose in Eq. (10) is a bit of approximation, based on the fitting curve $D_{\text{MSD}}(z)$ in Fig. 12; $C_{1}$, $C_{2}$ and $C_{0}$ are the fitting constants shown in Fig. 12.]

The CBCT dose in the Lucite phantom can be transformed to water phantom as follows

(11) $$D_{w}=D_{m}\left[\frac{{\left({\mu_{en/}}_{\rho }\right)}_{\text{air}}^{w}}{{\left({\mu_{en/}}_{\rho }\right)}_{\text{air}}^{m}}\right]$$

where $D_{m}$ is the dose measured in the Lucite phantom. ${\left(\mu_{en_{{/}_{\rho }}}\right)}_{\text{air}}^{m}$ the water‐to‐air ratio of the mean mass energy‐absorption coefficients averaged over the photon spectrum at the calibration depth.

### H. Anterior and lateral patient skin doses

The skin doses for seven pelvic tumor patients scanned with the CBCT Varian OBI system (v1.4) were measured using OSL nanoDots. The nanoDots for skin dose measurements were calibrated against the ionization chamber using the in‐air protocol from TG‐61,^(24)^

(12) $$D_{w,z=0}=MN_{k}NB_{w}{\left[{\left({\bar{\mu_{en}}}_{/\rho }\right)}_{\text{air}}^{w}\right]}_{\text{Air}},$$

where *M* is the free‐in‐air chamber reading, $N_{K}$ the air‐kerma calibration factor for the given beam's quality, and $B_{w}$ is the backscatter factor, which accounts for the effect of the water scatter.

The OSLDs were placed on the anterior skin and the left lateral and right lateral skin of the patients. The CBCT imaging system was operated using a standard clinical protocol for prostate tumor patients (125 kVp, 80 mA, and 25 ms). The half‐bowtie filter with a full 360° rotation and a 16‐cm scanning length (superior–inferior) was used for all scans. The irradiated OSL nanoDots were read using the same procedure as discussed in the Materials and Methods section E. The measured doses were tabulated against the patient thickness along the anterior–posterior and lateral directions.

## III. RESULTS

### A. CBCT dose measurements

#### A.1 Dose at the phantom surface

**Figure 3:**
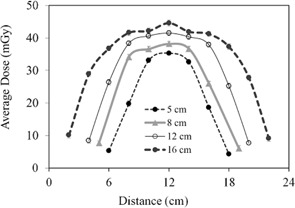
CBCT surface dose profiles of the body phantom along the superior–inferior direction for four FOVs.

**Figure 4:**
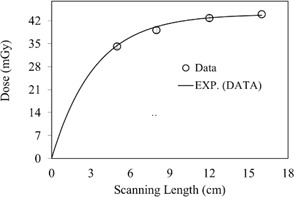
CBCT surface dose at the middle of the FOV as a function of scanning length (from Fig. 3). The symbol represents the data and the solid line is the exponential fitting curve. Fitting constants and $R^{2}$ values are listed in Table 3.

The CBCT dose at the phantom surface as a function of the superior–inferior position is shown in Fig. 3 for several FOVs. The dose profiles in Fig. 3 show a nonlinear increase as the FOV increases. This increase is illustrated more clearly in Fig. 4. The surface dose at the center of the FOV (scanning length) increased from 35.3±0.75 to 44.5±0.81 mGy as the FOV increased from 5 to 16 cm (Fig. 4). A plot of the measured central point doses against field size illustrates the increase in scatter dose (Fig. 4). From the exponential mathematic model (Eq. (6)), the equilibrium in scatter occurs at scanning length of 40 cm. This can be calculated using Eq. (6) and the fitting constants from Table 1.

**Table 1:** Patients' skin doses measured during CBCT (360° rotation with half‐bowtie filter at 125 kVp, current 80 mA and exposure time 25 ms). Seven prostate patients were scanned.

*Patient's #*	*Anterior–Posterior Thickness (cm)*	*Lateral Thickness (cm)*	*Anterior Skin Dose (mGy)*	*Lateral Skin Dose (mGY)*
1	16	36	48.48±1.24	28.15±1.19
2	19	37	38.98±1.18	27.67±1.11
3	20	38	37.94±1.38	24.43±1.05
4	21	38	37.94±1.15	27.91±1.21
5	21	39	38.32±1.05	27.91±1.24
6	22	39	37.57±1.02	26.97±1.19
7	23.5	40	34.12±1.01	26.61±1.22

#### A.2 Dose at the phantom center

**Figure 5:**
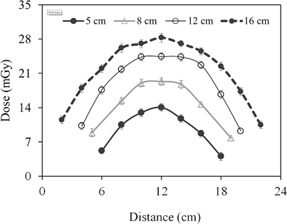
CBCT dose profiles at the center of the body phantom along the superior–inferior direction for different FOVs.

**Figure 6:**
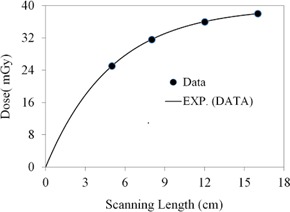
CBCT dose at the center of the body phantom (from Fig. 4) as a function of scanning length. The symbol represents the data and the solid line is the exponential fitting curve. Fitting constants and $R^{2}$ values are listed in Table 3.

The CBCT dose as a function of the superior–inferior position at the phantom center is shown in Fig. 5 for several FOVs. The dose was maximal at the isocenter and decreased rapidly in both the superior and inferior directions. The dose at a position 5 cm off the isocenter (superior or inferior direction) was reduced by 14%. This dose reduction is primarily attributed to the amount of scatter, which is focused at the isocenter. Additionally, the dose profiles in Fig. 5 showed a rapid increase as the FOV increased. These observations are illustrated in Fig. 6. The dose at the center of the field increased from 14.0±0.61 to 28.3±0.73 mGy as the FOV increased from 5 to 16 cm. In Fig. 6, the point‐dose equilibrium due to scatter occurs at scanning length of 43 cm.

#### A.3 Slice depth dose (profile along the rim)

**Figure 7:**
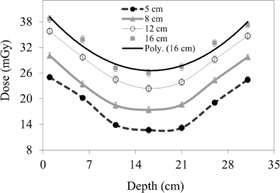
CBCT depth‐dose profiles of the body phantom along the diameter/rim.

**Figure 8:**
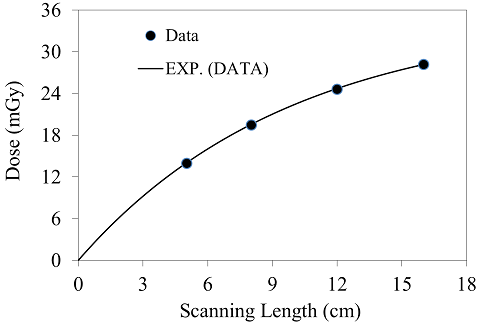
Peripheral dose as a function of scanning length (from Fig. 7). The symbol represents the data and the solid line is the exponential fitting curve. Fitting constants and $R^{2}$ values are listed in Table 3.

In Fig. 7, CBCT dosimetry results for the central slice are expressed as a function of nanoDot position and FOV. The CBCT doses at all measurement positions on the 32‐cm diameter of the body phantom increased with FOV as a second‐order polynomial, is shown in 7, 8. The measured doses at the center and periphery of CBCT acquisition at 125 kVp were 26.1±0.69 mGy and 38.48±0.78 mGy, respectively. As expected, the dose profile along the rim of the slice increases nonlinearly as a function of FOV. This is attributed to the amount of scatter generated in the phantom. The measured peripheral dose increased by a factor of 1.54 when the FOV was increased from 5 to 16 cm (Fig. 8). In Fig. 8, the point‐dose equilibrium due to scatter occurs at scanning length of 41 cm.

It can be noticed that almost no heel effect is evident in Fig. 7, because the anode axis of the OBI system is perpendicular to the axis of rotation; the heel effect is smeared out by 360° rotation of the CBCT scan.

**Figure 9:**
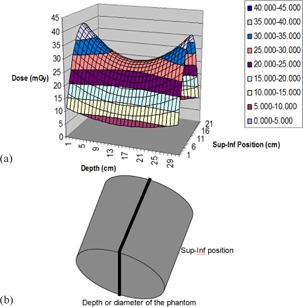
Two‐dimensional dose profile (a) of the sagittal plane obtained from the fitted data curves; (b) schematic of the body phantom, representing the sagittal plane.

The two‐dimensional dose profiles (or the distribution for the sagittal plane obtained from the fitting equations) are shown in Fig. 9. The dose decreases in both directions (superior and inferior) away from the isocenter, at all depths. However, the dose profile along the slice rim showed a minimum dose value at the slice center for all slices.

#### A.4 ${\text{CBDI}}_{w}$, mean slice dose, and mean volume dose

**Figure 10:**
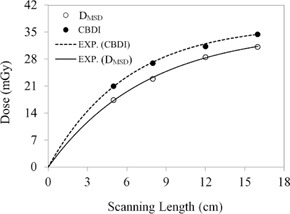
Mean slice dose, $D_{\text{MSD}}$, and CBDI dose plotted as a function of FOV, the solid and dotted lines representing the exponential fitting curve for $D_{\text{MSD}}$ and CBDI, respectively. Fitting constants and $R^{2}$ values are listed in Table 3.

**Figure 11:**
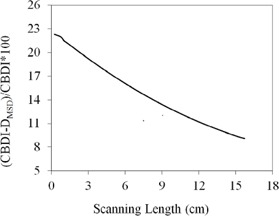
Percentage difference between CBDI and $D_{\text{MSD}}$ as a function of FOV (from Fig. 10).

The ${\text{CBDI}}_{w}$ dose calculated from Eq. (7) and the mean slice dose, $D_{\text{MSD}}$, calculated from Eq. (8) show a nonlinear increase as a function of FOV (Fig. 10). For a 16‐cm field, $D_{\text{MSD}}$ was 31.1±0.85 mGy, 12% smaller than the ${\text{CBDI}}_{w}$ dose of 34.5±0.76 mGy. The percentage difference between ${\text{CBDI}}_{w}$ and $D_{\text{MSD}}[({\text{CBDI}}_{w}-D_{\text{MSD}})/{\text{CBDI}}_{w}\times 100]$ as a function of scanning length is more clearly illustrated in Fig. 11.

**Figure 12:**
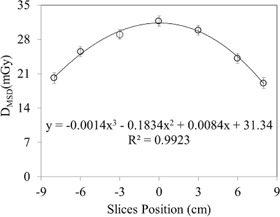
Mean slice dose profile as a function of superior–inferior position (cm). The symbol represents the mean slice dose for the corresponding slices location and line represents the polynomial fit.

The mean slice dose, $D_{\text{MSD}}(z)$, as a function of slice position (Z) is plotted in Fig. 12. $D_{\text{MSD}}$ is seems to be maximum for the central slice or at the middle of the scanning length. This is primarily attributed to the body scatter being higher at the center as well as the traveling length in the phantom. The solid line represents the data fitting curve. The fitting equation and the corresponding fitting constants are shown in Fig. 12. The $D_{\text{MVD}}$ (the mean volume dose) calculated from Eq. (10) for a 16‐cm FOV was 25.6±1.1 mGy, smaller by ∼5 mGy than the $D_{\text{MSD}}$ of the central slice (calculated from Eq (8)). It is illustrated in the point‐dose profile along the superior–inferior direction in Fig. 5, which shows high dose at the central slice position.

In Table 2, the ratio of CBDI (measured in the body phantom) to therapeutic doses of organs at risk (OAR) per fraction quantified from the prostate plans (integrated boost technique VMAT) is shown. The radiotherapy doses include maximum dose ($D_{\text{RT},\max}$) and mean dose ($D_{\text{RT},\text{mean}}$) to OAR extracted from the RapidArc treatment plans are shown in Table 2. The relative (%) CBDI to therapeutic doses of OAR[(CBDI/DRT,max)×100% and (CBDI/DRT,mean)×100%] are within 2% and 3%, respectively. The use of CBCT for prostate patient using integrated‐boost VMAT technique is adequate for daily patient alignment due to tight PTV margin. The relative CBCT doses to skin are comparatively higher than to the rest of OAR in Table 3.

**Table 2:** CBCT doses relative to radiotherapy doses per fraction to organ at risk (OAR) averaged over 10 prostate patients (extracted from the Eclipse RapidArc treatment plans). Integrated boost technique was used for all the prostate patients. The prescribed dose per fraction was 243 cGy.

*OAR*	*Rectum*	*Bladder*	*Sigmoid*	*Hip*	*Anterior Skin*
$D_{\text{RT},\max}$ per fraction (cGy)	238.8	244.1	188.2	157.4	24
$D_{\text{RT},\text{mean}}$ per fraction (cGy)	107.1	175.2	159.3	99.5	‐
$(\text{CBDI}/d_{\text{RT},\max})\times 100\%$	1.29	1.25	1.64	1.91	12.92
$(\text{CBDI}/D_{\text{RT},\text{mean}})\times 100\%$	2.89	1.77	1.94	3.11	‐

**Table 3:** Fitting constants and $R^{2}$ values for exponential mathematical Eq. (6).

*Figure #*	*A*	*B*	$R^{2}$
4	44	27 mm	0.996
6	35	98 mm	0.999
8	39.6	50 mm	0.998
10	37	60 mm	0.997
13	11.5	70 mm	0.993

#### A.5 Dose outside the FOV

**Figure 13:**
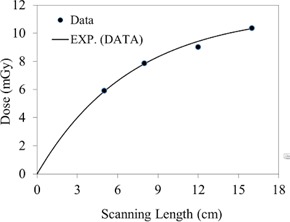
Dose outside the FOV, plotted as a function of scanning length at 16 cm depth in the phantom. The solid curve represents the exponential fitting curve. Fitting constants and $R^{2}$ values are listed in Table 3.

The point dose at a position 2 cm outside the field edge, plotted as a function of FOV, is shown in Fig. 13. The point dose at the phantom center increased from 5.96 to 10.6 mGy as the FOV changed from 5 to 16 cm, as illustrated in Fig. 13. Assuming that the scatter from the air and the collimator is negligible, this increase is primarily attributable to scatter from the phantom. Significant doses (42% and 23% of the total integral dose due to scattered radiation) were observed at distances 2 cm and 6 cm from the primary radiation FOV, at 16 cm depth. These results suggest that for a 16‐cm beam or FOV, the dose is largely due to scattered X‐rays that originate within the phantom and travel in all directions, transversely and longitudinally.

### B. Anterior and lateral skin doses of pelvic tumor patients

**Figure 14:**
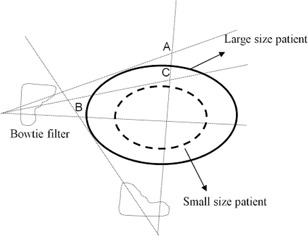
Schematic illustrating how bowtie filtration varies with patient diameter. In the patient measurements, the half‐bowtie filter was used; the half bowtie in the figure is just for illustration. The skin dose at point A depends upon the bowtie filtration from the lateral beam and anterior exiting dose. The dose at point B is expected to be less than dose at point A, because of the large lateral thickness and bowtie filtration.

The skin doses at the anterior and lateral locations for CBCT scans of seven patients are shown in Table 1. The average doses at the anterior and lateral skin were 39.04±4.4 and 27.1±1.3 mGy, respectively. The variation in anterior skin doses among the patients (at point A in Fig. 14) was higher than that of the lateral skin doses (at point C), which is attributed to variation in the anterior–posterior patient's thickness, that caused different bowtie filtration from the lateral direction and exiting anterior dose. The average anterior–posterior thickness is less than the lateral thickness by a factor of approximately 1.8 (Table 1), that's why the anterior–posterior skin doses are higher than the lateral skin. The details regarding the bowtie filtration in terms of patient or phantom thickness has been discussed by Mail et al.^(24)^

## IV. DISCUSSION

The OSL nanoDot dosimeter showed excellent dose linearity and reproducibility when benchmarked against an ionization chamber. It is worth mentioning that when using the OSL dosimeter for in‐phantom dosimetry, the application of depth‐dependent correction factors is highly recommended to account for the energy dependence of the dosimeter due to variations in beam quality at increasing depths (i.e., as a result of beam hardening and the addition of a scatter component). The need for depth‐dependent correction factors was evaluated, as written in Eq. (3), and it was found that the sensitivity of the dosimeter varied by only 8.5% from surface to center of a 32 cm diameter phantom. Much of this variation occurred between the free‐in‐air measurement (depth=0) and the calibration point at a depth of 2 cm in the phantom (Fig. 2(b)), suggesting that the OSL dosimeter could be calibrated only to a depth of a few cm into the scattering medium to minimize the effect of the dosimeter's energy dependence for in‐phantom dose measurements. It should be noted that specific irradiation geometries and conditions should be evaluated to determine the extent to which changes in the beam energy spectrum could affect the dosimeter's calibration. We have analyzed the scatter and the primary component of the kV X‐ray beam in CBCT scans at several depths, and our results illustrate that the scatter component is almost three times larger than the primary beam at 16 cm depth or center. The impact of such a huge Compton scatter for a large body phantom reduces the beam hardening issues as a function of depth.

It is important to note that nanoDots in each new batch should be selected for minimum variations in exposure detection or quantum detection efficiency, to ensure high reproducibility. As stated earlier, in CBCT dosimetry the effect due to energy variations will likely be averaged out for in‐phantom measurements, due to the rotation of the X‐ray tube. We found that the effect due to energy for OSL nanoDots is slightly lower than the single radiographic projection. The energy sensitivity of the nanoDots was verified against an ionization chamber, calibrated at 125 kVp.

The cone beam dose index, ${\text{CBDI}}_{W}$, calculation from Eq. (7) shows a 12% higher dose than the mean slice dose, $D_{\text{MSD}}$, calculated from Eq. (8). The $D_{\text{MSD}}$ was calculated from large data points in the slice integrated over the slice volume, while CBDI is based on the two point measurements; definitely, $D_{\text{MSD}}$ is consider to be more accurate compared to ${\text{CBDI}}_{W}$. This study does not recommend the use of $D_{\text{MSD}}$ for periodic QA purposes as $D_{\text{MSD}}$ calculation procedure is more time‐consuming, but it definitely give us a fundamental knowledge and understanding regarding the level of approximation in ${\text{CBDI}}_{W}$. The CBCT dose profiles along the superior–inferior directions resulted in a 10%–14% reduction at positions ±5 cm from the isocenter. This confirms that the use of a 10‐cm‐long pencil chamber would provide an approximation as opposed to an actual measurement of the dose of each slice along the superior‐inferior direction. It is therefore difficult to resolve or evaluate the central slice dose with a 10‐cm pencil chamber. With nanoDots, it is possible to measure a point dose (which is maximum at the central slice), and this has some physical relevance for organs that could be at risk from exposure if CBCT is used every day. The DMSD for the central slice was expected to be higher than the DMVD, because of the higher scatter at the center of the body phantom. The volume integral dose from Eq. (10) (obtained from dose profiles and fitting equations) can be useful in quantifying the total energy deposited in the tumors and/or the organs at risk (OAR).

In literature, there are debates in favor of and against the use of CBCT‐IGRT system. It is well understood from the literature that there are identifiable risks attributable to radiation dose, which are likely to increase with the addition of the CBCT dose. However, in many cases, the likely benefits of performing IGRT using CBCT, including reduced CTV‐PTV margins and reduced geographic miss, justifies the use of CBCT for daily patient alignment. With the use of CBCT, we can achieve CTV2 to PTV2 margins of 3 mm without compromising tumor control.

In this study, the dose measured outside the CBCT scanning volume was reasonably high, which is very important in terms of OAR. The dose outside the field was analyzed and primarily attributed to scatter from the phantom. The large discrepancies between the lateral skin doses and the phantom are due to the large lateral thickness and the heterogeneous nature of the body, such as bony anatomy, that causes reduction in the exiting dose at the lateral skin and significant bowtie filtration. The small discrepancy between the phantom and the anterior skin doses is primarily attributed to the bony anatomy, causing reduction in the exiting anterior skin doses.

## V. CONCLUSIONS

The sensitivity of the dosimeter varied by only 2% with the application of depth correction factor (calibration of OSL against ion chamber as a function of depth). The nanoDot selection technique described in this manuscript was useful in terms of improving reproducibility and decreasing uncertainty in the measurements. The calibration procedure, and in particular the development of a new equation for nanoDot calibration, could be very helpful for the medical physics community. It has the potential to be a good substitute for conventional CTDI measurements using a 100‐cm pencil ionization chamber. The difference between $D_{\text{SMSD}}$ and ${\text{CBDI}}_{W}$ for the body phantom under full scatter conditions suggests that a minor modification is required in the calculation of ${\text{CBDI}}_{W}$ by Eq. (7). The volume integral dose obtained from nanoDots can provide useful information. NanoDots enable point‐dose measurements at any location on the phantom, including the surface, periphery, and center, and at any depth. Using OSL nanoDots, we managed to estimate the amount of scatter and primary doses during CBCT on a Varian OBI system. Owing to its high sensitivity and small size, nanoDot dosimetry could be used frequently in patient skin dose assessment without degrading or harming the image quality.

## ACKNOWLEDGMENTS

This project was funded by the National Plan for Science, Technology and Innovation (MAARIFAH), King Abdulaziz City for Science and Technology, the Kingdom of Saudi Arabia, award number (08‐MED113‐03). The authors also, acknowledge with thanks the Science and Technology Unit, King Abdulaziz University, for technical support.

## COPYRIGHT

This work is licensed under a Creative Commons Attribution 3.0 Unported License.
